# Factors associated with medication adherence of hypertensive patients in the Philippines: a systematic review

**DOI:** 10.1186/s40885-021-00176-0

**Published:** 2021-10-01

**Authors:** Margarita M. Gutierrez, Rungpetch Sakulbumrungsil

**Affiliations:** 1 University of the Philippines Manila College of Pharmacy, Manila, Philippines; 2 Faculty of Pharmaceutical Sciences, Chulalongkorn University, Bangkok, Thailand

**Keywords:** Hypertension, Medication adherence, Patient compliance, Pharmacy, Philippines

## Abstract

**Background:**

Diseases of the heart and vascular system are the leading cause of mortality in the Philippines. Hypertension, the most important modifiable risk factor, has a prevalence rate of 28% and a control rate of 20%. Despite the proven efficacy of pharmacologic treatment, medication adherence is reported to be as low as 66%. While there are publications that reported factors that affect adherence in Filipinos, there are no existing research that evaluated them systematically. This review is conducted to present and synthesize findings of published literatures.

**Methods:**

Databases—PubMed, Scopus, Wiley Online library, Science Direct, JSTOR, Web of Science, SAGE journals, and Cochrane—were used to search for articles published from 2000 to 2020 that studied medication adherence in adult Filipino hypertensive population. Out of the initial 1514 articles, 15 articles met the criteria and were included in the analysis. The evidence from the included studies was summarized and discussed in a narrative review using the World Health Organization framework for adherence to long-term therapies as the framework.

**Result:**

The factors that were positively associated with adherence were health care system-related factors: good patient-health provider relationship, accessibility of health services, use of specialty clinics and programs for hypertension, and health insurance. The factors found to be negatively associated with adherence are (1) social economic factors: younger age, single civil status, low educational attainment, and unemployment; (2) patient-related factors: low in health literacy and awareness, knowledge on hypertension, attitude towards hypertension, self-efficacy, and social support; (3) therapy-related factors: inconsistent drug regimen schedule, use of Thiazide and complementary and alternative medicines; (4) condition-related factors: low illness perception, and absence of comorbidities.

**Conclusions:**

Findings should be interpreted with caution because of methodological limitations. Despite this, given that health systems related factors are modifiable, they can be the focus of interventions and future researches to increase medication adherence. Clinicians may also want to screen their Filipino hypertensive patients for factors that are associated to low adherence in order to provide a tailored advice. Longitudinal research studies with heterogeneous samples of hypertensive Filipinos are imperative so that targeted interventions can be developed for the population.

## Background

Six of the ten leading cause of death in the Philippines are non-communicable in etiology, the leading cause being cardiovascular diseases [1]. A systematic review by Kearney et al. PM Kearney, M Whelton, K Reynolds, P Muntner, PK Whelton and J He [2] found that hypertension is a the most important modifiable risk factor for stroke and myocardial infarction that applies to both developed and developing countries. A prospective, multi-staged, stratified nationwide survey on hypertension was conducted in the Philippines found that the prevalence of hypertension is 28% or approximately 29 million citizens, and it is projected to increase over time [3–5]. According to Musini et al. VM Musini, AM Tejani, K Bassett and JM Wright [6], treating healthy persons (60 years or older) with moderate to severe systolic and/or diastolic hypertension with antihypertensive drug, reduces all-cause mortality and cardiovascular morbidity and mortality. Despite the proven efficacy of antihypertensive drugs in controlling blood pressure, patient adherence in clinical practice is reported to be as low as 20 to 50% and therefore it can be presumed that many patients, experience difficulty in following long-term treatment recommendations [7].

Adherence, as defined in chronic disorders context by the World Health Organization (WHO) [8] is “the extent to which a person’s behavior with respect to taking medication, following a diet, and/or executing lifestyle changes, corresponds with agreed recommendations from a healthcare provider”. High adherence is defined objectively as medication possession ratio of 80 to 100%. Historically, high adherence to hypertensive medications is associated with higher blood pressure control [9]. In 2007, Philippine Heart Association-Council on Hypertension Report on Survey of Hypertension showed treatment rates for hypertension is at 65%, however, of those receiving treatment the adherence rate is at 66% resulting to a hypertension control rates of only 20% [10]. This is consistent to the findings of de Guzman et al. [11] where they found out that the elderly Filipino patients have low adherence at only 41.54%.

While it is easy to assume that perhaps medicine access is the main barrier to adherence, there are findings that suggest that even Filipino immigrants in developed countries are also non adherent to hypertensive medication therapy, when compared to other Asian American counterparts despite the availability of medications [12–16]. Thus raising the question if there culturally or uniquely Filipino characteristics or factors that contribute to phenomenon of non-adherence beyond barriers of access. Moreover, the Philippine government in the last 10 years have medicine access programs to respond to the persistently high out of pocket spending and barriers to access to essential drugs [17–19]. While there are weaknesses in the implementation of these program it is still worth exploring if there are other factor beyond medicine access that contribute to medication adherence [19].

The purpose of this article was to critically review the literature on factors associated with medication adherence in hypertensive Filipinos. No such systematic review appears to have been previously conducted. The review may be used to recommend focus of future researches and propose interventions to improve it in hypertensive Filipino patients.

## Main text

### Data sources and search strategy

The search strategy was run on eight databases: PubMed, Scopus, Wiley Online library, Science Direct, JSTOR, Web of Science, SAGE journals, Cochrane library between the year 2000 to 2020. A facet analysis broke the review question into its component parts population (Filipino), intervention (for hypertension and anti-hypertensive) and outcome (medication adherence). Index terms such as ‘hypertension’ and ‘medication adherence’ were explored and all sub-headings were included. The resulting search term is ((((((“patient compliance”) OR “patient adherence”) OR “medication compliance”) OR “medication adherence”)) AND ((Antihypertensive) OR Hypertension)) AND ((Philippines) OR Filipino). Subsequently, the reference list of included studies were scrutinized to identify additional relevant studies. Corresponding authors were searched and contacted to include these literatures in the review as well.

### Study selection

**Fig. 1:**
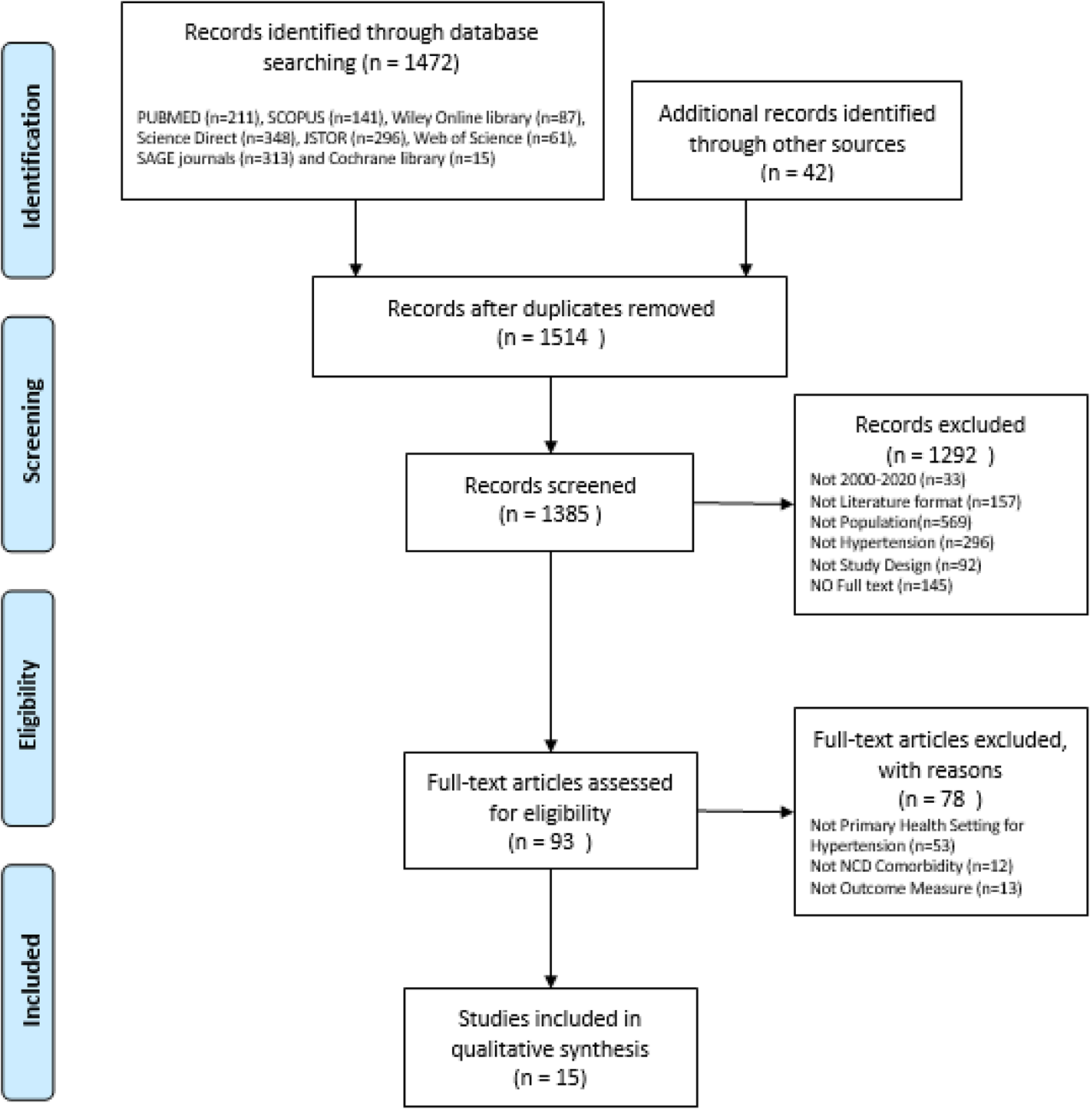
Flow diagram of the search strategy based on PRISMA

Study samples included in the review were individuals aged 18 years and above of Filipino ethnicity, with hypertension and receiving antihypertensive treatment from primary care, outpatient or community setting. The outcomes considered in the review was medication adherence that is measured using objective measures or validated subjective (self-reported) instruments, summarized in Table 1 [14, 16, 20–28]. Review articles, conference presentations and discussion papers were excluded. The process of selecting these studies are illustrated in Fig. 1 flow diagram of the search strategy based on PRISMA [29].

**Table 1:** Outcome measures for medication adherence

	Code	Description
Subjective measures (self-reported adherence)		
Hill-Bone high blood pressure compliance scale [20]	HB-HCT [20]	This scale uses a 14-item Likert scale to measure three behavioral domains a) reduced-sodium intake b) appointment keeping c) medication taking [20]. Cronbach’s alpha = 0.74 and 0.84.
Self-structured questionnaires (ten items) [21]	SSQ-10 [21]	Researcher created tool which was validated with 23 respondents who served as the pilot study of this research [21]. Cronbach’s alpha value = 0.7.
Morisky Medication Adherence Scale [22]	MMAS-8 [22]	MMAS-8 is a series of eight binary questions, a “No” is one point. A score of 8 indicates high adherence, 6–7 is medium, and < 6 is poor adherence [22]. Cronbach’s alpha = 0.83.
11 Items adapted MMAS-8 [23]	Adapted MMAS-8 [23]	10- to 11-item questionnaire with scores ranging from 0 to 44. Adherent have scores, 0 to 21 and non-adherent scores, 22 to 44 [23]. Cronbach’s alpha = 0.932.
Medical Outcomes Study Specific Adherence Scale [24]	MAOSS [24]	Assesses of participant’s tendency to adhere to eight behaviors associated with hypertension self-care that include patient’s ability to follow a salt and low fat or weight loss diet, take prescribed medications, cut down or stop smoking, curtail or avoid alcohol, exercise regularly, avoid stress, and use relaxation techniques for the past 4 weeks measured using a 6-point Likert scale. The higher the mean score, the greater the adherence [24]. Cronbach’s alpha = 0.811.
Binary adherence question	Binary [25]	Is the respondents taking the right medications at the right dosages at the right time, answerable by yes or no [25].
Medication Adherence Questionnaire [26]	MAQ [26]	A total score of 0–1 was defined as adherent while 2 or above was considered as non-adherent [26].
Adherence Self-Report Questionnaire [27]	ASRQ [27]	A brief self-administered questionnaire measuring “timing adherence,” defined as taking medications at the correct dose and intervals. Has six items describing adherence to the timing of medication intake. Adherence was defined as an ASRQ score of less than or equal to 2 [27]. Specificity 90.3% and sensitivity 14.6%.
Objective measures		
Proportion of days covered	PDC [14]	Days the patient was covered by at least one drug for each type of medication, based on the prescription fill date and days of supply divided by the number of days of drug coverage and multiplied by 100. Medication adherence was defined using the standard threshold of PCD greater than 80% [14]. Data on filled medications including medication names, fills and days of supply were obtained from pharmacy claims databases [14].
Medication Possession ratio	MPR [28]	Calculated by dividing the no of day supply dispensed by the no of days evaluated multiplied by 100%. For the research they used the formula Possession ratio = days supplied for 1stRX/(filldateof2ndRX-filldateof1stRX). Possession ratio of 0.8 or greater was considered adherent [16, 28]

HB-HCT: Hill-Bone High Blood Pressure Compliance Scale; SSQ-10: Self-structured questionnaires (ten items); MMAS-8: Morisky Medication Adherence Scale; MAOSS: Medical Outcomes Study Specific Adherence Scale; MAQ: Medication Adherence Questionnaire; ASRQ: Adherence Self-Report Questionnaire; PDC: Proportion of days covered; MPR: Medication Possession Ratio

### Data extraction and screening

All retrieved articles were organized and screened using a Microsoft Excel spread sheet and EndNote management software. After removing duplicates, articles’ titles and abstracts were assessed for eligibility. Then, the full-text articles were screened and data of the eligible articles were extracted into a standardized data extraction table. Extraction of the data was based on the following categories: research design, objective, study setting, sampling technique, sample size, eligibility criteria, measure of medication adherence, independent variables tested, medication adherence result, key findings and any statistical information (odds ratios, 95% confidence interval, *P*-value, correlation coefficients). Quality of the studies was evaluated and reported using the Strengthening the Reporting of Observational Studies in Epidemiology (STROBE) for observational studies, Consolidated Standards of Reporting Trials (CONSORT) 2010 for parallel group randomized trials and the Joanna Briggs Institute Checklist for Quasi-Experimental Studies.

### Data analysis

A meta-analysis of the findings was not possible due to the heterogeneity of important aspects of the methodology of the selected studies including sampling procedure, population ages, study settings, study design and measurement procedure of medication adherence. Descriptive analysis of studies examining similar variables and any association observed were considered to offer a simple indication of the level of evidence. Summary ranges of quantitative proportions and measures relating to prevalence and factors associated with medication adherence were compiled and presented on Table 1 [14, 16, 20–28] and Table 2 [11, 14–16, 21, 23, 25–27, 31, 34–38] The evidence from the studies was synthesized and presented in a narrative review following the WHO’s report on adherence as the framework of the review. In this framework, there are five interacting dimensions for medication adherence: (1) social/economic factors, (2) patient-related factors, (3) health team and system-related factors, (4) therapy-related factors, and (5) condition-related factors [8].

**Table 2:** Description of the studies

First author (year)	Source	Quality of research		Study design					
		Journal rank (SJR 2018) [30]	Score based on checklist	Research design	Objective	Population	Sampling technique	Filipino sample size	Eligibility criteria
**Calano (2019)** [31]	PubMed	Q1 (nursing)	JBI [32] (8/9)	Quasi-experimental (pre-test–post-test)	To determine the effectiveness of a community-based health program grounded on the knowledge, adherence and blood pressure control of adults with hypertension.	Adults with hypertension	Purposive sampling	50	**Inclusion:** 40–59 years old patients diagnosed with hypertension for at least 6 months from Bulacan, Philippines **Exclusion:** physical and/or mental disabilities
**Coyoca (2013)** [21]	Google Scholar	Not available	STROBE [33] (17/22)	Observational (cross-sectional)	To explore the barriers and factors that have an effect of the adherence to therapeutic regimen.	Adults with hypertension and type II diabetes	Convenience sampling	56	**Inclusion:** 18 years old and above patients diagnosed with hypertension and type II diabetes recruited from private and public clinics of Iligan City Philippines
**de Guzman (2013)** [11]	Research Gate	Q3 (education)	STROBE [33] (14/22)	Observational (cross-sectional)	To develop and test a model of medication adherence among Filipino elderly.	Geriatric patients with chronic illness (including hypertension)	Random sampling	325	**Inclusion:** > 60 years old, diagnosed with chronic illness, and with at least one long-term medication **Exclusion:** mental or cognitive disabilities
**Dror (2005)** [34]	Science Direct	Q1 (health policy)	JBI [32] (7/9)	Observational (cross-sectional)	To examine evidence of association between affiliation with Micro Insurance Units (MIU) and healthcare utilization.	Adult patients with chronic illness (including hypertension)	Cluster sampling method	504	**Inclusion:** households in sixMIUs from Northern Philippines, Metro Manila, Central Visayas and Mindanao, Philippines
**Ea 2018** [35]	Science Direct	Q2 (nursing)	STROBE [33] (18/22)	Observational (cross-sectional)	To explore self-care among Filipino immigrants in the United States who have hypertension.	Adult with hypertension (Filipino immigrants in the United States)	Convenience sampling	163	**Inclusion:** at least 18 years old, first generation Filipino immigrant, able to speak and write in English, and who had a current diagnosis of hypertension or were currently taking an antihypertensive medication at the time of recruitment **Exclusion:** pregnant or taking a contraceptive medication
**Encabo (2017)** [23]	CU Library	Q4 (pharmaceutical science)	STROBE [33] (2.5/9)	Observational (cross-sectional)	To know the percentage of the people who were adherent to their medication and know the factors that contributed to the medication adherence.	Adult with hypertension (member of hypertensive club)	Purposive sampling	94	**Inclusion:** at least 19 years old, diagnosed with hypertension for at least a month, and are either taking or prescribed with maintenance medications for hypertension from Caloocan, Philippines recruited from hypertensive club and barangay and the local municipality recommendation
**Juarez (2013)** [14]	Wiley Online Library	Q2 (economics, econometrics)	STROBE [33] (22/22)	Observational (retrospective analysis)	To identify factors associated with years of medication adherence and to examine the relationship between years of adherence and healthcare utilization.	Adult patients with diabetes and hypertension (Filipino immigrants in the United States)	Purposive sampling	1748	**Inclusion:** adult patients with diabetes who were enrolled in a large health plan in Hawaii for 4 years between 2007 and 2010 and at least one prescription medication
**Ku (2015)** [25]	SAGE Library	Q3 (health policy)	STROBE [33] (19/22)	Observational (cross-sectional)	To measure factors that could be associated with self-management practices of people with type 2 diabetes and hypertension.	Adults with hypertension and diabetes	Purposive sampling	549	**Inclusion:** at least 20 years old, diagnosed with type 2 diabetes and hypertension for at least six months, recruited from local government unit in Luzon, Philippines
**Mamangon (2018)** [26]	Grey literature	Not available	STROBE [33] (20/22)	Observational (cross-sectional)	To identify the proportion of the patients who are non-adherent and identify factors associated with medication adherence behavior.	Adults with hypertension	Convenience sampling	47	**Inclusion:** 25–59 years old diagnosed hypertensive in Barangay 898, Manila and recipients of free antihypertensive medication from J. Vicencio Health Center **Exclusion:** not able to speak in English or Filipino, advised to discontinue medications by physicians
**Pablo (2018)** [36]	Research Gate	Not available	JBI [32] (8/9)	Quasi-experimental (pre-test–post-test)	To determine the most prevalent complementary and alternative medicine (CAM) and determine if associated with medication adherence. To assess whether medication adherence seminar significantly increased the medication adherence.	Adults with hypertension and/or diabetes	Purposive sampling	66	**Inclusion:** at least 18 years old clinically diagnosed with hypertension and/or diabetes, currently prescribed with their maintenance medicine while practicing or using CAM, may or may not have not more than 2 comorbidities recruited from National Government Center, Quezon City, Philippines
**Palileo-Villanueva (2011)** [27]	Grey literature	Not available	STROBE [33] (18/22)	Observational (cross-sectional)	To determine the prevalence of adherence to blood pressure lowering medications and identify factors affecting it.	Adult with hypertension (outpatient specialty clinic)	Convenience sampling	276	**Inclusion:** patients with hypertension consulting at the General Medicine Outpatient Continuity Clinic of the Philippine General Hospital **Exclusion:** newly diagnosed to have hypertension or not yet maintained on antihypertensive medications at the time of the interview
**Taira (2006)** [15]	PubMed	Q1 (health policy)	STROBE [33] (22/22)	Observational (retrospective analysis)	To measure the impact of medication copayment level and other predictors on compliance with antihypertensive medications.	Adult with hypertension (members of a managed care organization)	Purposive sampling	13,708	**Inclusion:** diagnosed hypertensive with at least 1 antihypertensive medication prescription with at least a 15-day supply between January 1999 and June 2004
**Taira (2007)** [16]	PubMed	Q1 (arts and humanities)	STROBE [33] (22/22)	Observational (retrospective analysis)	To identify predictors of adherence for specific groups, particularly Asian Americans and Pacific Islanders.	Adult with hypertension (members of a managed care organization)	Purposive sampling	3812	**Inclusion:** 18 years old diagnosed hypertensive with drug coverage in a large health plan. Must have filled at least one prescription for one of five classes of identified antihypertensive medications. **Exclusion:** Patients using other therapeutic classes, including adrenergic inhibitors, alpha-adrenergic blocking agents, other diuretics or combination drugs and/or newly shifted to a different medication
**Ursua (2014)** [37]	PubMed	Q2 (medicine)	JBI [32] (8/9)	Quasi-experimental (pre-test–post-test with control)	To assess the feasibility and efficacy of a 4-month community health worker intervention to improve hypertension management among Filipino immigrants in New York and New Jersey.	Adult with hypertension (Filipino immigrants in Unites States)	Purposive sampling	88	**Inclusion:** 25–75 years old, self-identified as Filipino in New York City or Jersey City, US (had one systolic blood pressure (SBP) reading of 132 mmHg or one diastolic blood pressure (DBP) reading of 82 mmHg **Exclusion:** on renal dialysis, had participated in a previous cardiovascular disease (CVD) study, or had experienced a heart attack or stroke
**Ursua (2018)** [38]	Science Direct	Q1 (health informatics)	CONSORT [39] (19/25)	Randomized clinical trial	To assess the efficacy of the intervention on blood pressure control, SBP and DBP, and compliance to appointment keeping.	Adult with hypertension (Filipino immigrants in United States)	Random Sampling	182	**Inclusion:** 25–75 years old, self-identified as Filipino in New York city, and diagnosed hypertensive or on antihypertensive medication use **Exclusion:** on renal dialysis, had an acute or terminal illness or serious mental illness, had participated in a previous CVD study, or had a history of heart attack, stroke, or congestive heart failure

SJR, SCImago Journal Rank; JBI, Joanna Briggs Institute; STROBE, Strengthening the Reporting of Observational Studies in Epidemiology; CONSORT, Consolidated Standards of Reporting Trials

## Results

A total of 1472 citations from the databases were identified. After elimination of duplicate records and abstract screening, 93 articles were full-text reviewed. The reference list of the selected literatures was then scrutinized that resulted in the inclusion of 42 articles for further screening. The final number of articles included in this systematic review is 15: one randomized controlled trial [38], three quasi-experimental [21, 26, 36], three observational retrospective analysis [14–16], and eight observational cross-sectional study [11, 23, 25, 27, 31, 34, 35, 37]. The description of the included studies is summarized in Table 2. The cumulative number of study participants is 21,668 with age of ranging between ages 18 to 75 years old.

### Medication adherence

Of the 15 studies on Table 3 [11, 14–16, 21, 23, 25–27, 31, 34–38] only two studies [23, 27] reported a baseline medication adherence to be high, however in both researches, a subjective or self-reported adherence method is used for measurement. The rest of the studies reported low adherence, four [14–16, 34] of them utilize objective measure for medication adherence.

**Table 3:** Summary of findings

First author (year of study)	Measure of medication adherence	Independent variables tested	Medication adherence of Filipino sample	Statistically significant findings
Calano (2019) [31]	HB-HCT [20]	Not applicable	Pre-test 1.40 (8.56) Post-test 17.98 (5.74)	Community-based health program (*P*-value = 0.03) F-value = 5.00) [31]
Coyoca (2013) [21]	SSQ-10 [21]	1. Age 2. Gender 3. Religion 4. Civil status 5. Educational attainment 6. Work status 7. Family monthly income 8. Social support system 9. Length of diagnosis 10. Blood sugar level 11. Awareness of the disease 12. Relationship towards their doctors 13. Availability and accessibility to healthcare services 14. Consultation with consultation in public clinics	Values cannot be determined.	1. Gender (*P*-value = 0.033), female has higher adherence [21]. 2. Civil status (P-value = 0.016), married has higher adherence [21]. 3. Work status (P-value = 0.037), working patients has higher adherence [21]. 4. Social support system (Spearmen rho P-value = 0.028) – direct relationship [21] 5. Accessibility to health care services (Spearman rho P-value = 0.000) – direct relationship [21] 6. Consultation with consultation in public clinics (P-value = 0.016) – direct relationship [21] 7. Health care provider-patient relationship (P-value = 0.016) – direct relationship [21] 8. Health awareness (Spearman rho P-value = 0.000)- direct relationship (26)
de Guzman (2013) [11]	MMAS-8 [22]	1. Social support 2. Medication belief 3. Follow-up visits 4. Consultation satisfaction, 5. Memory task 6. Trust with physician 7. Perceived stress 8. Memory strategies 9. Memory load 10. Depression 11. Length of time taking the medications 12. Number of conditions 13. Self-efficacy	Low adherence 41.54% (*n* = 135) Medium adherence 39.69% (*n* = 129) High adherence 18.15% (*n* = 59)	1. Trust with physician (β = 1.168) – direct [11] 2. Consultation satisfaction (β = − 0.215) – inverse [11] 3. No. of conditions (β = 0.693) – direct [11] 4. No. of medication (β = − 0.151) – inverse [11] 5. Event-based memory(β = − 0.329) – inverse [11] 6. External memory strategy (β = − 0.186) – inverse [11] 7. Depression (β = 0.215) – direct [11]
Dror (2005) [34]	Compliance (binary question taking drugs prescribed)		Uninsured chronically ill not taking drugs is 32.6% while insureds is lower at 20.2%.	Insured persons reported better drug compliance among chronically ill (P-value = 0.0015) [34].
Ea (2018) [35]	MAOSS [24]	1. Acculturation 2. Acculturative stress 3. Hypertension self-efficacy 4. Patient activation		1. Self-efficacy (by self-efficacy scale) (P-value = 0.003) (β = 270) – direct 2. Patient activation P-value = 0.024) (β = 205) – direct
Encabo (2017) [23]	Adapted MMA8 [23]	1. Age 2. Gender 3. Marital status 4. Employment 5. History of hypertension 6. Presence of other diseases 7. Medication	14.5 (SD = 13.6), with scores ranging from 0 to 44 (scores of 22 to 44 is non adherent) 77.7% (*n* = 73) patients were adherent and 22.3% (*n* = 21) are non-adherent.	Use of maintenance drugs (*P* = 0.016) based on odds ratio (OR)
Juarez (2013) [14]	PDC [14]	1. Years of adherence 2. Healthcare utilization	Mean years of adherence for antihypertensive medication is 2.17 years.	1. Age older was significantly associated with greater adherence 1.33 (1.26, 1.42) [14]. 2. Female sex was significantly associated with fewer years of adherence 0.9 (0.89, 0.99) [14]. 3. Ethnicity Filipino negatively associated with adherence. OR 0.90 (080–1.00) [14] 4. Comorbidities history of either coronary artery disease 1.19 (1.11, 1.28) or congestive heart failure 1.20 (1.09, 1.32) was significantly associated with more years of adherence to antihypertensive medications [14]. 5. Poly pharmacy being on lipid-lowering 1.38 (1.28, 1.49) and antidiabetic medications 1.34 (1.26, 1.43) increased adherence on antihypertensive.
Ku (2015) [25]	Binary [25]	1. Age 2. Knowledge 3. Attitudes 4. Perceptions of support 5. Perception of self-efficacy 6. Obesity/adiposity 7. Specialty clinic 8. Body mass index (BMI) 9. Waist circumference, and waist–hip ratio		1. Age (P = 0.002) older more adherent [25] 2. Specialty clinic had better adherence [25] (Fisher’s exact test *P* < 0.001) [25]. 3. Knowledge (*P* = 0.007) higher more adherent [25] 4. Positive attitude (P < 0.001) higher more adherent [25] 5. Perception of support (P < 0.001) higher more adherent [25] 6. Perception of self-efficacy (*P* = 0.004) higher more adherent
Mamangon (2018) [26]	MAQ [26] and MMAS-8 [22]	1. Presence of comorbidities 2. Illness perception 3. Patient-doctor relationship 4. Health literacy	51.06% were non adherent to antihypertensive medication, 61.70% have forgotten to take antihypertensive medicine during the last two weeks.	1. Age aged 25–59 years old were non-adherent to antihypertensive medication [26]. 2. Patient-doctor relationship (PR, 1.6; 95% confidence interval [CI], 0.96–2.75) associations but not statistically significant [26]. 3. Comorbidity (PR, 1.15; 95% CI, 0.66–2.01) associations but not statistically significant [26]. 4. Illness perception associations but not statistically significant (PR, 1.61; 95% CI, 0.50–5.17) [26]. 5. Health literacy (PR, 1.96; 95% CI. 0.90–4.27) associations but not statistically significant [26].
Pablo (2018) [36]	Adapted MMAS-8 [23]	1. Attitude towards complementary and alternative medicine (CAM) 2. Medication adherence seminar	Mean score pre-intervention period is 2 (mean = 1.8939; SD = 0.86164) equivalent to “Sometimes” non-adherent to their medication.	1. Post-seminar intervention (*P* = 0.000), increase in the medication adherence of patients [36] 2. A significant negative correlation between medication adherence and CAM Attitude Pearson correlation r value = − 0.730, *P*-value = 0.049), increase in medication adherence if low CAM attitude. Inverse relationship [36]
Palileo-Villanueva (2011) [27]	ASRQ [27]	1. Socioeconomic factors a. Sex b. Age c. Employment status d. Economic status 2. Condition related a. Chronicity of hypertension b. Blood pressure (BP) c. BP control d. Number of comorbidities e. Comorbidities 3. Therapy related a. Number of medications b. Number of anti c. Class of antihypertensive medications d. Cost – weekly cost of antihypertensive medications 1. Patient factors a. Educational attainment b. Knowledge scores	Adherence is 72% in a specialty clinic.	1. Number of maintenance medications increasing has more adherence increase in the number of drugs a patient has, the odds of being more adherent increases by 1.15 times (*P* = 0.05) [27]. 2. Financial support from children, patients that were being supported by their children were twice more likely to be adherent (*P* = 0.002) [27].
Taira (2006) [15]	MPR [28] and binary	1. Age 2. Gender 3. Ethnicity 4. Morbidity level, health plan type 5. Isle of residence 6. Comorbidities 7. Year of treatment 8. Physician ethnicity	Adherence rates were less than 65% among all racial/ethnic groups.	1. Ethnicity Filipino patients were least adherent, compared to whites (*P* < 0.001) [15]. 2. Age lower adherence in younger age (P < 0.001) [15] 3. Educational attainment. Adherence improved with increase (P < 0.001) [15]. 4. Patients seeing cardiologists or other specialists were less adherent than patients seeing primary care doctors (P < 0.001) [15]. 5. Patients seeing female physicians were less adherent than those seeing male physicians (P < 0.001) [15]. 6. Patients seeing Filipino physicians tended to be less adherent than patients seeing white physicians (*P* < 0.001) [15]. 7. Patients with a history of diabetes tended to be more adherent (P < 0.001) [15]. 8. History of heart disease has lower adherence (P < 0.001) [15]. 9. Adherence to be highest for beta blockers and calcium channel blockers, followed by Angiotensin receptor blocker and Angiotensin converting enzyme inhibitors. Adherence to all these therapeutic classes was significantly higher than adherence to thiazide diuretics (P < 0.001) [15].
Taira (2007) [16]	MPR [28]	1. Copayment level 2. Age 3. Ethnicity 4. Morbidity level 5. Therapeutic class	Age low compliance, age 40 years (42.5% compliance), 40 to 64 years were nearly twice as likely to be compliant with medications [16]. Ethnicity low compliance, Filipino ethnicity (58.7% compliance) [16]	1. Low compliance members wit Health maintenance organization coverage (59.7% compliance). Members of HMOs had lower compliance than members of preferred provider organization [16]. 2. Copayment level, independent of other determinants, was found to be a strong predictor of compliance with antihypertensive medications (*P* < 0.05) [16]. 3. Greater compliance seen among patients filing pharmacy claims for drugs that required lower copayments [16]. 4. Compliance was lower for drugs in less preferred tiers [16]. 5. Lower medication compliance was seen in those patients with high morbidity (i.e., indicating the presence of other comorbid conditions) compared with patients with low comorbidity [16]. 6. Best compliance observed for angiotensin receptor blockers, followed by calcium channel blockers, [beta] adrenergic receptor antagonists ([beta]-blockers), angiotensin-converting enzyme inhibitors, and last, thiazide diuretics [16]. 7. Filipino patients were more likely than other ethnic groups to have received tier 3 (for medications with a $20 to $165 copayment most expensive) medications (13.4% vs. 12%) [16].
Ursua (2014)	HB-HCT [20]		Pre-test 11.15 (2.73) Post-test 11.54 (2.15)	CHW intervention significant changes were exhibited for systolic and diastolic BP, weight, and BMI (*P* < 0.01) but not significant for medication adherence.
Ursua (2018)	HB-HCT [20]		Pre-test intervention 3.6 (0.5) vs. control 3.6 (0.5) P-value = 0.867 Post-test intervention 3.8 (0.5) vs. control 3.7 (0.3)	1. Community-based intervention delivered by CHWs improve BP and related factors [38]. 2. Adjusted odds of controlled BP for the treatment group was 3.2 times the odds of the control group (P < 0.001) and individuals in the treatment group showed significant changes in appointment keeping [38]. 3. Weight, and BMI improvement (P < 0.01) [38]

HB-HCT, Hill-Bone high blood pressure compliance scale; SSQ-10, Self-structured questionnaires (ten items); PR, Prevalence ratio; MMAS-8, Morisky Medication Adherence Scale; MAOSS, Medical Outcomes Study Specific Adherence Scale; SD, standard deviation; PDC, proportion of days covered; MAQ, Medication Adherence Questionnaire; ASRQ, Adherence Self-Report Questionnaire; MPR, Medication Possession ratio; CHW, community health worker

### Social/economic factors

The systematic review identified eight studies in which social/economic factors from the WHO adherence model was examined. Eight studies examined the association between age and medication adherence; Five of the studies determined that there is a significant negative association between younger age and adherence rates [14–16, 25, 26]. Three of the five use retrospective analysis and objective measures of medication adherence [14–16]. In two of the studies the age group below 40 years old is twice less likely to be adherent when compared to 40 to 64 years old [16, 26]. It should be noted however that three studies that use cross-sectional study design did not find an association between age and medication adherence [21, 23, 27].

Four studies examined the association between gender and medication adherence; two of them determined that there is a significant association while the other two stated that they found none. The conclusions of both studies are inconsistent, in terms of which sex (male or female) has lower adherence [14, 21]. Overall, the result is in inconclusive.

Only one study examined the association of civil status, they determined that in sample of 56 participants married female participants has higher adherence [21]. Two studies examined if the educational attainment impacts the medication adherence; Taira et al. [15] determined that adherence improved with increased educational attainment, however in both Coyoca et al. [21] and Palileo-Villanueva et al. [27] no association was found. Coyoca et al. [21] found an association between work and adherence where working patients has higher adherence.

### Patient-related factors

Patient-related factors are the knowledge, attitudes, beliefs, and perceptions of hypertensive patients [8]. There are six literatures that examined patient-related factors. Patient’s health literacy has positive relationship but not statistically significant in the study conducted by Mamangon et al. [26]. Disease awareness on the other hand is statistically significant in the study of Coyoca et al. [21] where they related this to the coping mechanism to stress of the participants. In two literatures, knowledge is directly associated with medication adherence [25, 35]. For attitude, three studies concluded that positive attitude lead to higher adherence [11, 25, 35].

Result of studies on self-efficacy is mixed, one of the studies did not find an association, but in two of them concluded that higher patient’s perceived self-efficacy is associated with higher adherence [11, 25, 35]. In the case of social support, three studies found a direct relationship between social support system and higher adherence [21, 25, 27]. Specifically, in the study of Palileo-Villanueva et al. [27], they specifically found that patients that were being supported by their children financially were twice more likely to be adherent.

### Health team and system-related factors

Healthcare team and system-related factors include patient provider relationship and logistical barriers to healthcare [8]. Accessibility to health services and insurance whether locally or abroad showed positive relationship with adherence. Locally, Coyoca et al. [21] and Dror et al. [34], statistically proven the link where insured persons reported better drug compliance among chronically ill. Consistent findings were observed in Filipinos residing abroad, they determined that copayment level, independent of other determinants, was found to be a strong predictor of compliance with antihypertensive medications specifically when offered by the patient’s preferred organization. In addition, greater compliance was seen among patients filing pharmacy claims for drugs that required lower copayments. They also found an inverse relationship with compliance to medications in the most expensive tiers [16].

Four literatures examined the effect of patient and health care provider relationship, Coyoca et al. [21] determined that there is a positive relationship between good relationship and adherence, this is also consistent with the findings of Mamangon et al. [21, 26]. In the de Guzman et al. [11]‘s study, an interesting finding is that higher trust with physician resulted to higher adherence, but curiously if the patient has high satisfaction with the doctor consultation the adherence tend to be low. In Taira et al. [15]‘s study, a study conducted abroad, found out that adherence was higher if patients are consulting with primary care doctors over specialist. Filipino patients also tend to have lower adherence with a female and Filipino physicians.

In three studies, consultation in specialty continuity clinics for hypertension showed positive relationship with adherence whether it is public or privately managed institution [21, 25, 27]. The same positive relationship is observed in the four community-based health programs researched, regardless whether they are a short term (2-h lecture) or long-term (6 months program). There is an observed increase in adherence post-intervention in these researches [31, 36–38].

### Therapy-related factors

Therapy-related factors include the complexity of the medical regimen, duration of treatment, frequent changes in treatment, and adverse effects [8]. The findings with regards to number of medications or polypharmacy is mixed in three of the studies; de Guzman et al. [11], found that the number of medication is inversely proportional with medication adherence. But in two of the studies, it appears that the increasing number of medication led to higher adherence. In Palileo-Villanueva et al. [27], they specifically stated that the odds of being more adherent increases by 1.15 times if the patient has polypharmacy. In Juarez et al. [14], they emphasized that poly pharmacy on lipid-lowering and antidiabetic medications are statistically significantly increased adherence on antihypertensive.

Despite the two latter findings that associated polypharmacy and adherence positively it should be noted that, certainly confounders, such as multiple comorbidities, advance stage of illness, and patient’s illness representation among others may have contributed to this. In other words, the statement “odds of being more adherent increases by 1.15 times if the patient has polypharmacy” should have be more accurately reported as “patients in the advance stages of their illness with multiple medications are more adherent by 1.15 times”. In order to validate the result and to avoid hasty conclusions, it is recommended for future researchers that when studying the association of adherence and polypharmacy, instead of running a univariate analysis it would be better to use multivariate regression models in order to account for potential sources of bias from confounders. This recommendation is consistent with studies conducted in other countries where it has shown repeatedly that fewer medications improved adherence.

In Encabo et al. [23], the use of maintenance drugs that means consistent scheduling of medication had a positive relationship with adherence. An interesting findings of de Guzman et al. [11] appeared that Filipino elderly preferred no assistance in remembering medication intake whether using event-based memory or external memory based strategy. In Taira et al. [15], they suggested that adherence is highest for beta blockers and calcium channel blockers, followed by angiotensin receptor blockers and angiotensin-converting enzyme inhibitors and that adherence is lower in thiazide diuretics. This finding is somewhat consistent with Taira et al. [16], thiazide diuretics also has the lowest adherence, but in this paper best compliance observed for angiotensin receptor blockers, followed by calcium channel blockers, beta blockers, angiotensin-converting enzyme inhibitors in that order. With regards to CAM, Pablo et al. [36] found that medication adherence is higher if the patients has low attitude towards CAM.

### Condition-related factors

Condition-related factors are those illness-related demands faced by the patient, such as severity of blood pressure, absence or presence of symptoms, and level of disability and/or comorbidities [8]. For the number of comorbidities, it appears that the finding for Filipinos abroad have different pattern compared to patients locally. In the finding of de Guzman et al. [11] and Mamangon et al. [26] as the number of other conditions increased, adherence also increased. But in Taira et al. [16], a study conducted abroad, lower medication compliance was seen in those patients with high morbidity.

As for the type of comorbidity, Juarez et al. [14] determined that history of either coronary artery disease or congestive heart failure was significantly associated with more years of adherence to antihypertensive medications. The result conducted abroad however, showed an opposite relationship. For Taira et al. [15], the history of heart disease has lower adherence but history of diabetes tended to be more adherent.

## Discussion

The result of medication adherence is consistent with the national survey reported in PRESYON2 that the adherence among those treated for hypertension is 66% [10]. The factors found that are consistently associated with adherence across the included studies were all health systems related. The factors that increase adherence are (1) accessibility of health services, (2) positive relationship with providers of health services, (3) specialty clinics and programs for hypertension, and (4) health insurance. This is comparable to the finding in the United States, where patients reporting better access to health care, satisfaction with their care and a good patient-doctor relationship were significantly more likely to be more adherent [40].

The number of studies conducted exploring reasons for non-adherence in hypertensive Filipinos is limited and majority are also cross-sectional which makes it difficult to assess causality. Further, we don’t know if the relationships are sustained over time. Despite these limitations, given that health team and system-related factors are modifiable, these can be the focus of interventions and further research to increase medication adherence.

**Fig. 2:**
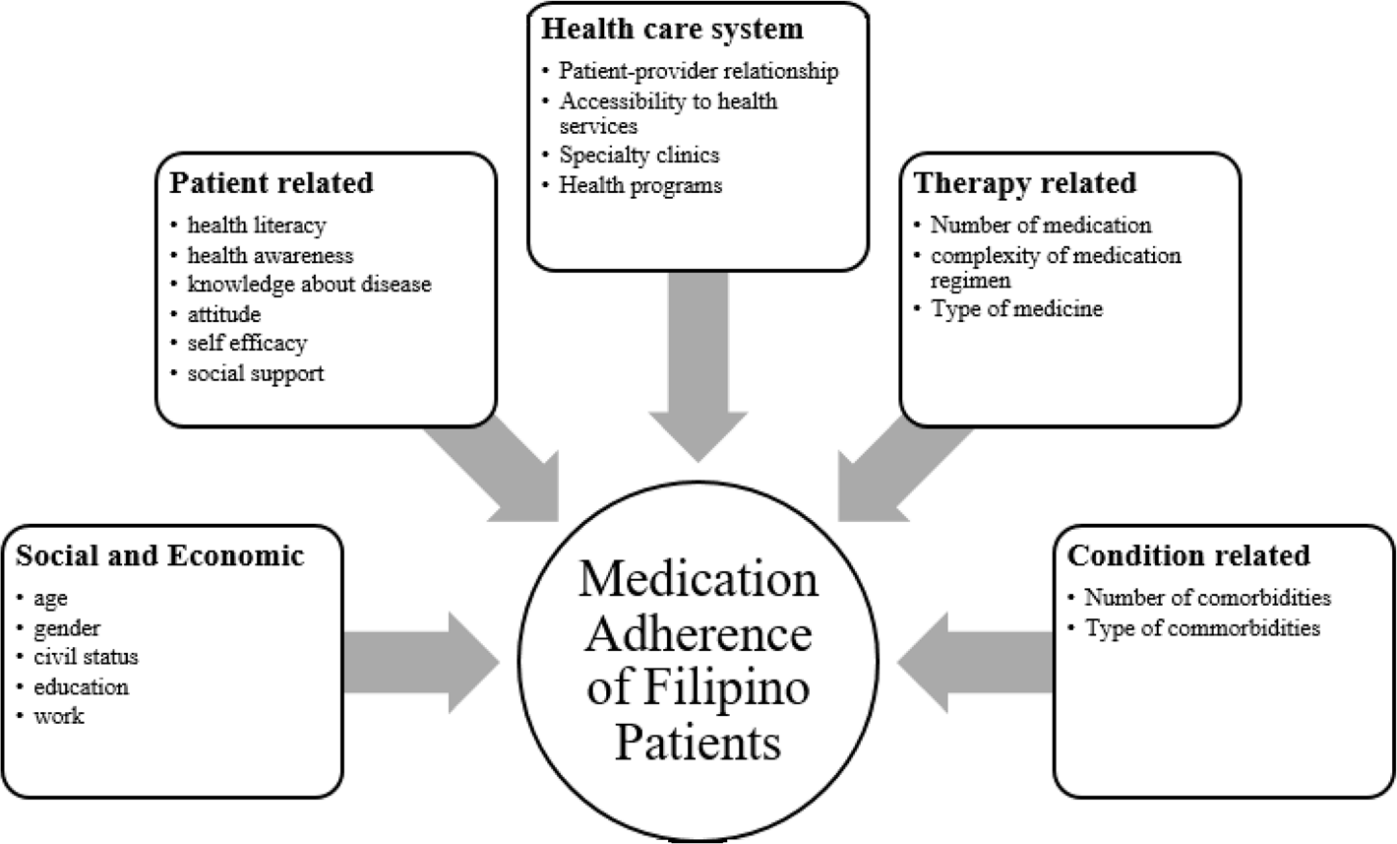
Factors that are associated with Adherence in Filipinos

The other four dimensions of medication adherence, have patterns of association as well, but are not consistent in all included studies. The factors that are associated to poor adherence are younger age, single in civil status, low educational attainment, unemployment, low health literacy and awareness, low knowledge on hypertension, negative attitude towards hypertension, low self-efficacy, low social support, inconsistent drug regimen schedule, taking Thiazide diuretics, taking CAMs, low illness perception, and no comorbidities as seen in Fig. 2. Factors that are associated with Adherence in Filipinos These findings, must be interpreted with caution because of the small number of studies and the fact that most were cross-sectional as well. Healthcare providers may still consider screening for these issues when dealing with their hypertensive patients.

This review revealed that more researches are needed to determine which factors influence antihypertensive medication adherence in Filipinos. Several concerns were identified after this systematic review, first concern regarding these studies is that most of the studies were based on homogeneous samples of hypertensive Filipinos, often they are even collected within the same locale or they are recruited from the same organization or health insurance providers. This will therefore limit generalizability. Another concern in interpreting the results of this review is that most of the studies measured medication adherence using subjective methods or by self-report, and there are inherent biases when using this tool. Consistent objective measurements of adherence are lacking and need to be incorporated as an outcome for future studies when exploring factors associated with medication adherence. The third concern is related to research design, since adherence is a multidimensional and dynamic process, it cannot be assumed that medication adherence and factors associated with medication adherence are stable over time. Most of the studies exploring medication adherence were observational and cross-sectional. There is a need for more longitudinal analyses of factors associated with medication adherence so that we can determine if an important factor remains associated with adherence at different time periods across a patient’s hypertensive diagnosis. Finally, there are other factors and interventions for enhancing medication adherence in hypertension like unit dose dispensing, combination pills, use of technology for dosing reminders, and blood pressure monitoring that have not been examined for their associations to medication adherence in Filipinos.

## Conclusions

Because of the limited number and methodological limitations of included studies exploring the factors associated with medication adherence in hypertensive Filipino patients, no definitive conclusions could be made about associations. Despite this, given that health systems related factors are modifiable, they can be the focus of interventions and future researches to increase medication adherence. Review suggests that continued support and enhanced government access programs and specialty clinics for Filipino hypertensive patients is crucial to achieving better adherence rates.

In addition, clinicians must also strive to develop positive relationships with their patients and when necessary, screen for the factors related to non-adherence and adjust counselling techniques accordingly. For future researchers, more information is needed to determine the factors associated with antihypertensive medication adherence for Filipinos. Effort must be done to continue finding them through longitudinal studies that preferably use with objective instruments to measures adherence. These researches is needed so that targeted interventions for medication adherence can be further developed that will ultimately improve mortality rates among Filipino patients with hypertension.

## Data Availability

Data sharing is not applicable to this article as no datasets were generated or analyzed during the current study.

## Abbreviations

ASRQ: Adherence Self-Report Questionnaire
CAM: Complementary and alternative medicines
HB-HCT: Hill-Bone High Blood Pressure Compliance Scale
MAOSS: Medical Outcomes Study Specific Adherence Scale
MAQ: Medication Adherence Questionnaire
MMAS-8: Morisky Medication Adherence Scale
MPR: Medication Possession Ratio
PDC: Proportion of days covered
SSQ-10: Self-structured questionnaires (ten items)
WHO: World Health Organization
